# Nickel in agri-food systems: a review

**DOI:** 10.3389/fpls.2026.1829209

**Published:** 2026-06-26

**Authors:** Alessio Elia, Onofrio Davide Palmitessa, Pietro Santamaria

**Affiliations:** Department of Soil, Plant and Food Sciences, University of Bari Aldo Moro, Bari, Italy

**Keywords:** food safety, hydroponic cultivation, nutrient solution, systemic nickel allergy syndrome (SNAS), vegetables

## Abstract

Nickel (Ni) is a widely distributed element in environmental and agri-food systems, deriving both from geogenic sources, such as ultramafic rocks and serpentine soils, and from anthropogenic inputs related to industrial activities, fossil fuel combustion and phosphate fertilization. Its ubiquitous presence favors its transfer into terrestrial ecosystems and the food chain, where plants represent the main interface between environmental compartments and the human diet. In the plant system, Ni plays a biphasic role: it is an essential micronutrient, acting as a cofactor of urease and involved in nitrogen metabolism, but it becomes phytotoxic at relatively high concentrations, inducing oxidative stress, photosynthetic alterations and nutritional imbalances. Absorption, transport, and accumulation in tissues depend on species, genotype, soil and environmental conditions, and agronomic practices, with direct implications for content in consumer products. In humans, exposure occurs predominantly via the dietary route; in sensitized individuals, even low concentrations can cause systemic manifestations, including systemic dermatitis and systemic Ni allergy syndrome (SNAS). The adoption of Regulation (EU) 2024/1987 introduced binding maximum limits for Ni in several food categories, strengthening the protection of the general population. In parallel, the increasing diffusion of “Ni-free” and “Ni-tested” products highlights an emerging demand, in the absence, however, of harmonized criteria for the use of such claims. In this context, the term “Ni-free” is generally used to refer to products with Ni concentrations below the detection or quantification limits of the analytical method used. Despite the extensive literature available, there remains a significant gap in the integration of environmental, agronomic, and regulatory perspectives to quantitatively identify the main factors governing Ni transfer along the soil–plant–food continuum. This review analyzes Ni transfer pathways from environmental sources to plant systems, the food chain, and human exposure, integrating physiological, agronomic, toxicological, and regulatory aspects. Particular attention is paid to closed-loop soilless systems, in which the Ni content depends on technical inputs and not on soil geochemistry. The results of the mass balance analysis indicate that mineral fertilizers can account for more than 80% of the total Ni input in the nutrient solution (NS), while pesticides and growing media play a secondary role. Overall, this review provides an integrated framework for understanding Ni transfer across agri-food systems, highlighting the central role of agronomic inputs in determining Ni accumulation in crops. These findings have direct implications for agricultural management and food security and support the development of reliable and standardized approaches for “Ni-free” production systems.

## 1 Introduction

Nickel (Ni) is a transition metal widely distributed in the environment as a result of both natural geochemical processes and anthropogenic activities. Naturally, Ni is released from ultramafic rocks through weathering and soil-forming processes, leading to elevated concentrations in specific geological contexts (Ghaderian et al., 2007; Quantin et al., 2008). At the same time, industrial emissions, fossil fuel combustion, metallurgical activities, phosphate fertilizers, and urban waste streams have significantly increased Ni inputs into soils, water bodies, and the atmosphere, amplifying its environmental dispersion (Cempel and Nikel, 2006; Nziguheba and Smolders, 2008; Reimann and de Caritat, 2012). In recent years, Ni has emerged as a growing concern in the food system due to increased anthropogenic inputs, advances in analytical techniques, and growing attention to dietary exposure, particularly among vulnerable populations.

**Figure 1:**
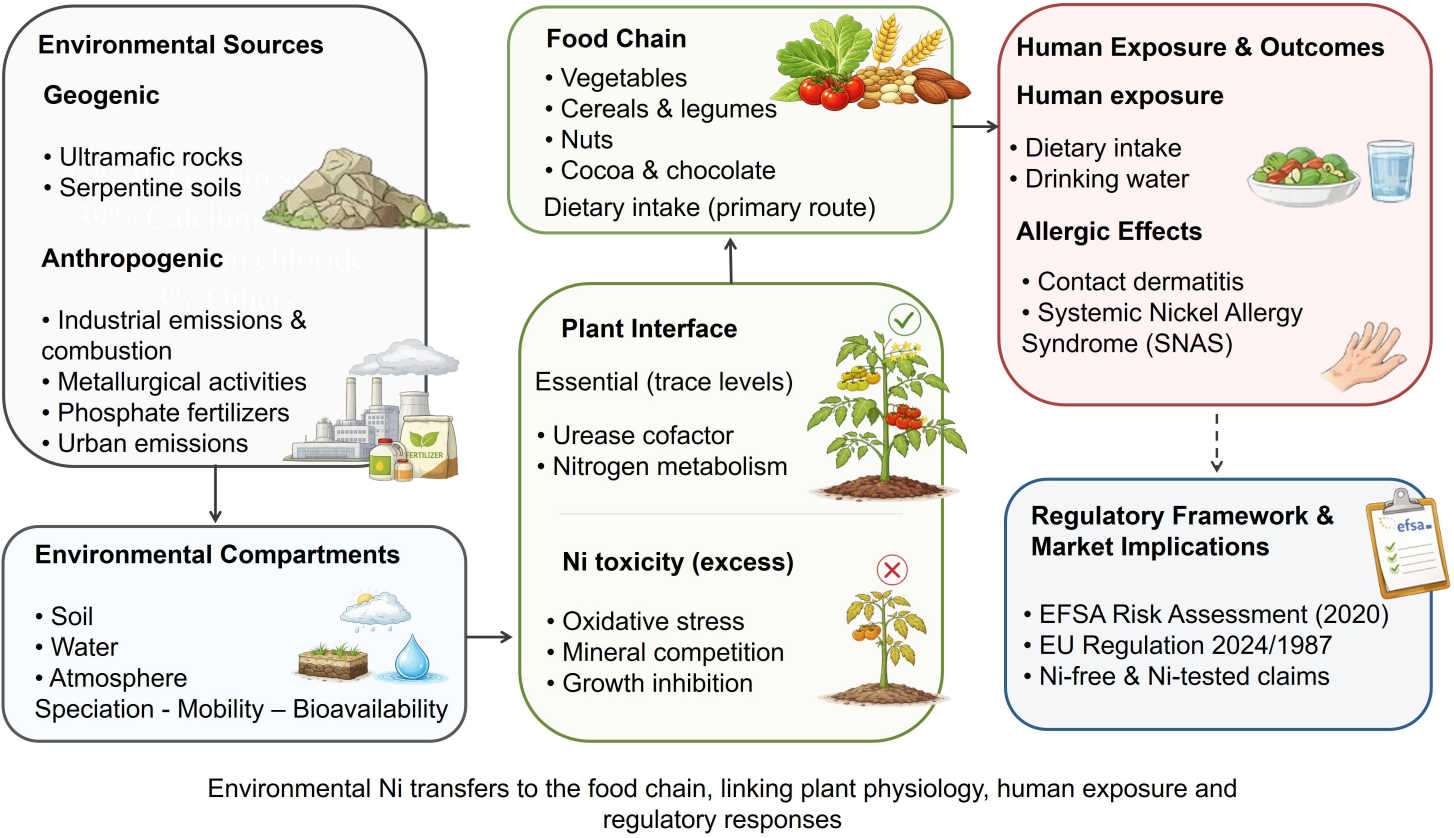
Conceptual framework of nickel transfer from environmental sources to the food chain and human exposure.

As a consequence of this widespread presence, Ni readily enters terrestrial ecosystems and the food chain. Plants represent a critical interface in this process, as they uptake Ni from soils in conventional cultivation systems and from nutrient solution (NS) in soilless systems, accumulating it in edible tissues depending on environmental conditions, agronomic practices, and species-specific traits (Chen et al., 2009; Gall et al., 2015). Numerous plant-based foods, including leafy vegetables, legumes, cereals, nuts, cocoa and chocolate products, are recognized as major dietary sources of Ni, contributing substantially to human exposure (European Commission, 2024; Pizzutelli, 2011; Schrenk et al., 2020). The interconnected pathways linking environmental Ni sources, plant uptake, transfer to the food chain, and human exposure are schematically summarized in **Figure 1**, describing the linkage between environmental sources, plant uptake, and human exposure.

Ni occupies a peculiar position among trace elements, because of its dual biological role. In plants, Ni is an essential micronutrient required in trace amounts for key physiological processes, particularly as a cofactor of urease and for efficient nitrogen metabolism (Dixon et al., 1975; Gerendás, 1999; Seregin and Kozhevnikova, 2006). However, when present at relatively elevated concentrations, Ni becomes phytotoxic, impairing germination, growth, photosynthesis, mineral nutrition, and redox homeostasis, ultimately reducing biomass and crop productivity (Bhalerao et al., 2015; Hassan et al., 2019; Shahzad et al., 2018).

In humans, dietary intake represents the main route of Ni exposure. Although gastrointestinal absorption from food is generally limited, bioavailability increases under specific conditions, such as fasting or consumption of drinking water, and Ni is recognized as one of the most common contact allergens (Ahlström et al., 2019; Schrenk et al., 2020). In sensitized individuals, even relatively low dietary intakes may trigger systemic reactions, including systemic contact dermatitis (SCD) and SNAS, characterized by gastrointestinal and dermatological symptoms (Duda-Chodak and Blaszczyk, 2008; Falagiani et al., 2008). Despite the extensive literature on Ni in environmental systems, previous reviews have often addressed individual aspects in isolation, with little integration across the soil to plant to food to human continuum. Furthermore, the emerging topic of “Ni-free” and “Ni-tested” products, as well as the role of Ni sources in controlled cropping systems, where agronomic inputs are the primary determining factor, remain underexplored.

Recent regulatory developments, such as the introduction of binding maximum levels of Ni in several food categories under Regulation (EU) 2024/1987, reflect increasing attention to chronic dietary exposure and consumer protection (European Commission, 2024a). Nevertheless, regulatory limits are primarily designed to safeguard the general population and do not fully address the growing demand for foods with particularly low Ni content, especially among Ni-sensitive individuals. This has contributed to the proliferation of voluntary claims such as “Ni-free” or “Ni-tested”, whose scientific basis, comparability, and regulatory status remain insufficiently defined. This regulatory and market context highlights the need for an integrated analysis of Ni behavior in agri-food systems. In this context, a comprehensive and integrative analysis of Ni behavior in environmental systems, plants, the food chain, and human health is needed. The present review aims to synthesize current knowledge on the sources, uptake, transport, and biological effects of Ni, with particular emphasis on its essential and toxic roles in plants, its transfer along the food chain, and its implications for human exposure and sensitivity. In particular, this review aims to integrate environmental, agronomic, toxicological, and regulatory perspectives, with a focus on identifying the main pathways and control points governing Ni transfer along the agri-food chain. By integrating physiological, agronomic, toxicological, and regulatory perspectives, this work seeks to provide a coherent framework for understanding the challenges associated with Ni in modern agri-food systems and to identify key gaps and future research directions. Particular attention is also paid to closed-cycle soilless cultivation systems, where Ni inputs are not regulated by soil geochemistry but by technical agronomic sources. For this reason, greater emphasis is being placed on soilless cropping systems, which represent a rapidly developing research area and a strategic platform for controlling Ni inputs and accumulation in crops. In such contexts, the reconstruction of the Ni mass balance through the quantitative analysis of fertilizers, plant protection products, substrates and irrigation water allows to identify the main entry routes and operational control points. This perspective is particularly relevant for the production of “Ni-free” and “Ni-tested” vegetables where even trace inputs can influence final concentrations in edible tissues. To address these aspects, the review follows the pathway of Ni across agri-food systems, from environmental sources to plant uptake, transfer along the food chain, and human exposure, and finally examines agronomic control strategies and the development of “Ni-free” production systems. However, available studies often report heterogeneous and sometimes inconsistent results, particularly regarding the relative contribution of environmental versus agronomic sources and patterns of Ni accumulation between plant species and cropping systems. This highlights the need for a more critical and integrative interpretation of existing evidence.

## 2 Sources of nickel in the environment

Ni distribution in environmental compartments is governed by geological background, industrial emissions, and agricultural practices. Geogenic sources are primarily associated with ultramafic rocks such as peridotites, serpentinites, and dunnites, which are rich in ferromagnesian minerals. Weathering and pedogenetic processes release Ni from minerals such as olivine and spinel, leading to elevated concentrations in derived soils. Serpentine soils may contain between 500 and 10,000 mg Ni·kg^−1^, values markedly higher than those of non-ultramafic soils (Ghaderian et al., 2007; Quantin et al., 2008). The mobility and bioavailability of Ni in these systems are strongly influenced by climatic conditions, redox processes, and soil pH (Chen et al., 2009).

Anthropogenic inputs further increase environmental Ni levels. Major sources include:

- industrial activities and fossil fuel combustion, which contribute to the atmospheric release and deposition of Ni-containing particles (Cempel and Nikel, 2006; Shahzad et al., 2018);
- metallurgical processes, particularly those associated with stainless steel and Ni alloy production (Cempel and Nikel, 2006);
- agricultural inputs, especially phosphate fertilizers derived from Ni-bearing phosphate rocks, which may contain on average up to 14.8 mg Ni·kg^-1^ (Nziguheba and Smolders, 2008);
- urban emissions related to traffic and waste disposal or incineration processes (Cempel and Nikel, 2006; Shahzad et al., 2018).

In soils, it is important to distinguish between the total Ni concentration and the bioavailable fraction, which represents the portion actually available for uptake by plants. While total Ni provides an estimate of the overall metal content, bioavailable Ni is determined by the soil’s physicochemical properties, such as mineralogy, iron and manganese oxides, pH, cation exchange capacity, and organic matter content. Under acidic or disturbed conditions, a larger fraction of Ni may become bioavailable, increasing the risk of uptake by plants and subsequent transfer into the food chain (Chen et al., 2009; Harasim and Filipek, 2015). Among these factors, soil pH plays a primary role, as decreasing pH increases Ni solubility and mobility. Organic matter also influences Ni mobility through complexation processes. These factors ultimately control the dynamic equilibrium between total and bioavailable Ni in environmental systems (Chen et al., 2009; Harasim and Filipek, 2015).

Given the combined influence of natural and anthropogenic sources, the distribution of Ni in environmental systems is highly variable and strongly influenced by both geochemical and anthropogenic factors. To facilitate comparison between these sources, **Supplementary Table 1** provides a summary of the main natural and anthropogenic inputs of Ni, including available data on concentrations and key characteristics. Sustainable management strategies are therefore necessary to limit environmental dispersion and biological exposure. Among remediation approaches, phytoremediation has emerged as an ecological and economically viable option. Hyperaccumulator species such as *Alyssum bertolonii Desv*. and *Berkheya coddii Roessler* are capable of accumulating more than 1,000 mg Ni·kg^−1^ dry biomass without exhibiting toxicity symptoms. In field conditions, *A. bertolonii* has been reported to yield up to 72 kg Ni·ha^−1^ through phytomining processes (Keeling et al., 2003; Robinson et al., 1997).

Field trials conducted on serpentine soils in regions such as Tuscany Italy and Albania have demonstrated the technical feasibility of Ni phytoextraction. The efficiency of the process can be enhanced through soil amendments, including nitrogen fertilization and selective chelating agents, which increase Ni mobility and plant uptake (He et al., 2012; Robinson et al., 1997). Harvested biomass may subsequently be processed for metal recovery, offering a sustainable alternative to conventional mining.

## 3 Nickel in the food chain

Ni enters the food chain through several pathways, affecting both plant and animal organisms and ac-cumulating progressively in different trophic levels. The main sources and the mechanisms of transfer and accumulation are described below, with a focus on human dietary exposures.

Ni can enter the food chain through natural and anthropogenic processes. Natural sources include re-lease from Ni-rich rocks and soils, erosion, and volcanic activities, while anthropogenic sources include industrial discharges, chemical fertilizer application, and urban and industrial wastes (Iyaka, 2011; Rizwan et al., 2024).

**Figure 2:**
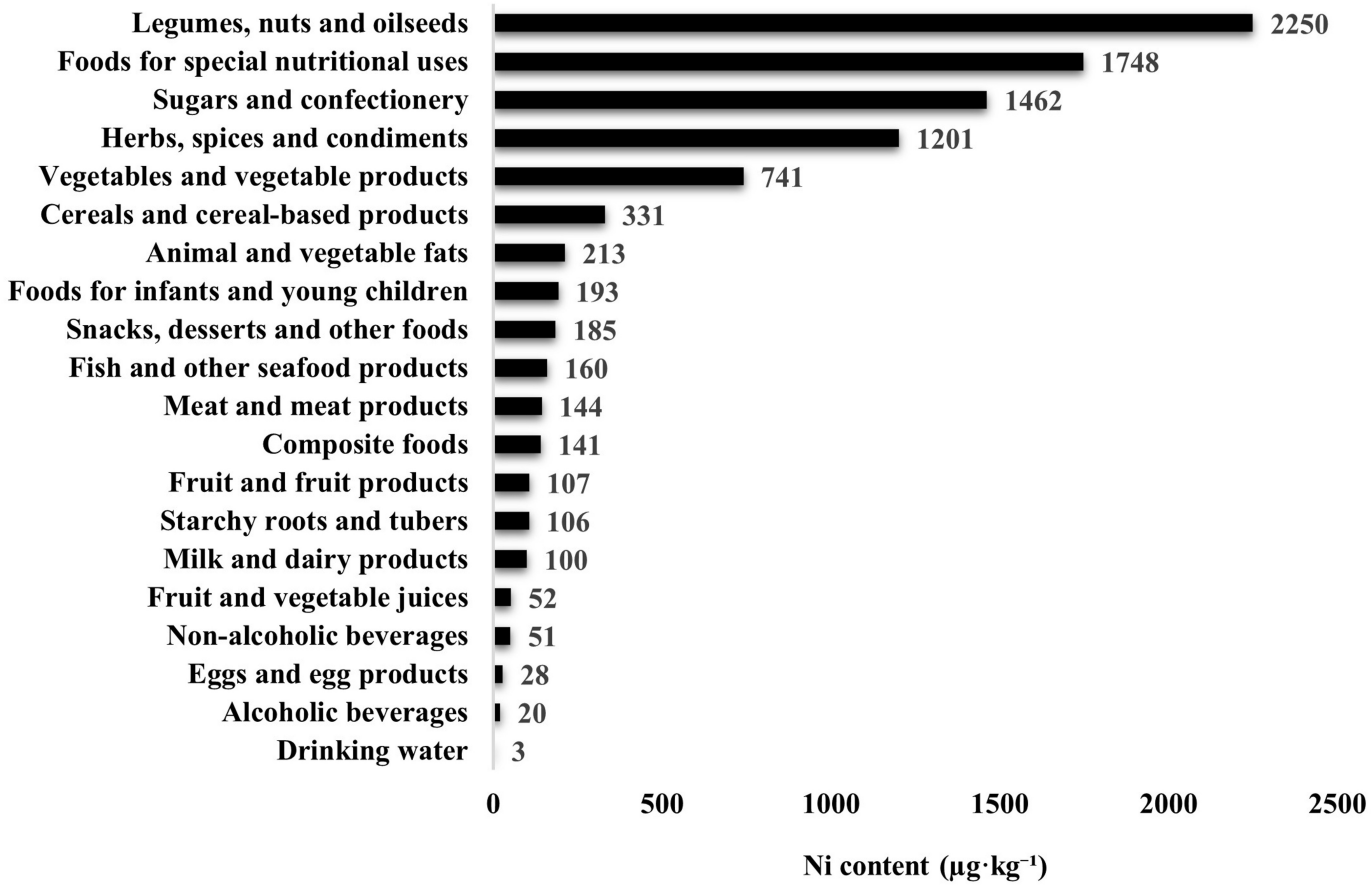
Average nickel content across major food categories, based on EFSA occurrence data (Schrenk et al., 2020).

Plants uptake Ni from soil, particularly in areas with high contamination, through water and nutrients necessary for their growth (Gall et al., 2015). The main food sources of Ni come from plants and plant products, which tend to accumulate the metal in their tissues. For example, root vegetables such as carrots, potatoes, and onions accumulate Ni when grown in contaminated soil or irrigated with water containing the metal (Stasinos et al., 2014). Similar phenomena have been reported in rice cultivated in paddy fields (Rahman et al., 2018). Vegetables such as spinach, legumes, and whole grains are particularly prone to Ni accumulation, as well as cocoa and some types of nuts (Schrenk et al., 2020). The presence of the metal in foods also depends on agricultural practices and soil quality (European Commission, 2024a; Schrenk et al., 2020). This variability is reflected in the average Ni content reported for different food categories, as shown in **Figure 2** reflecting the uneven distribution of Ni among food categories.

Once uptaken by plants, Ni can enter terrestrial food webs through trophic transfer. Herbivores ingest Ni by consuming contaminated plant tissues, and secondary consumers may further accumulate the metal through predation. Although biomagnification of Ni is generally limited compared to other heavy metals, its transfer across trophic levels may still contribute to chronic exposure in animals and humans (Gall et al., 2015). The extent of transfer depends on several factors, including Ni speciation, soil bioavailability, plant accumulation capacity, and physiological traits of exposed organisms (Iyaka, 2011; Schrenk et al., 2020).

In humans, dietary intake represents the primary route of exposure. Major contributors include green leafy vegetables (e.g., spinach and lettuce), legumes, nuts and oilseeds, cocoa and chocolate products, and whole grains (European Commission, 2024; Pizzutelli, 2011). In contrast, fresh fruits generally contain lower Ni concentrations and are not considered major contributors to dietary Ni intake. Ni concentrations in these foods are influenced by soil characteristics, agronomic practices, and environmental contamination.

Drinking water constitutes an additional and potentially critical source of exposure, particularly in contaminated areas. Reported concentrations typically range from 1 to 10 µg Ni·L^−1^, although higher values may occur near industrial sites (Ahlström et al., 2019). The European Union has established a maximum limit of 20 µg Ni·L^−1^ in drinking water. However, metal leaching from plumbing materials, especially under acidic or stagnant conditions, may increase concentrations (Nielsen et al., 1999). Importantly, Ni from drinking water shows substantially higher gastrointestinal bioavailability than Ni ingested with food, making water monitoring particularly relevant for risk assessment. Additional exposure may also arise from migration of Ni from food-contact materials; while high-quality stainless steel generally contributes negligibly, poor-quality alloys may release measurable amounts of Ni into food matrices. These exposure pathways have been discussed in detail in the EFSA risk assessment (Schrenk et al., 2020).

## 4 Effects of nickel on humans

Humans Ni absorption occurs primarily through the gastrointestinal tract and, to a lesser extent, through the skin. Its bioavailability is strongly influenced by chemical speciation and physiological conditions (Buxton et al., 2019). According to EFSA 2020, intestinal absorption is generally low (0.7–2.5%) when Ni is ingested with food but may increase to 25–27% under fasting conditions or following intake through drinking water (Schrenk et al., 2020). Once absorbed, Ni binds to serum proteins such as albumin and transferrin, facilitating systemic distribution. It can cross the placental barrier via active transport mechanisms, leading to fetal exposure and potential developmental toxicity (Italian Ministry of Health, 2016). Renal excretion represents the main elimination pathway (Blanc and Moreau, 2011).

At high doses, Ni exposure has been associated with hepatic and renal toxicity, alterations in body weight, bone metabolism, and changes in gut microbiota composition. Experimental animal studies report reproductive toxicity, including increased post-implantation loss and reduced offspring weight (Italian Ministry of Health, 2016); in addition, alterations in sperm motility and DNA integrity in males and increased risk of abortion and fetal malformations in females have also been reported (Rizvi et al., 2020; Schrenk et al., 2020). In sensitized individuals, even low exposures may trigger allergic responses, ranging from SCD to SNAS, characterized by gastrointestinal and dermatological symptoms (Duda-Chodak and Blaszczyk, 2008).

Ni is among the most prevalent contact allergens, affecting approximately 15–20% of the population, with higher prevalence in women (Falagiani et al., 2008). Sensitization is mediated by Ni-specific T lymphocytes, and repeated exposure may exacerbate immune responses. Although controlled oral desensitization protocols have been explored, standardized clinical guidelines remain limited (Duda-Chodak and Blaszczyk, 2008).

From a genotoxic perspective, Ni can interfere with DNA repair pathways and promote oxidative stress through increased reactive oxygen species (ROS) production, leading to single-strand DNA breaks and epigenetic alterations (Das et al., 2019; Genchi et al., 2020). While oral carcinogenicity remains inconclusive, Ni compounds are classified as Group 1 carcinogens by IARC based on evidence from inhalation exposure (Buxton et al., 2019).

Regulatory measures address Ni exposure in selected contexts. Regulation (EC) No 1223/2009 prohibits the intentional addition of Ni in cosmetic products, allowing only technically unavoidable trace amounts. More recently, Regulation (EU) 2024/1987 established maximum permitted levels of Ni in specific food categories to reduce chronic dietary exposure (European Commission, 2024a). However, specific dietary management guidelines for SNAS remain insufficiently defined.

Overall, the health impact of Ni depends on dose, duration, exposure route, and individual susceptibility. Further research is required to refine risk assessment models, particularly concerning bioavailability, immunological mechanisms, and vulnerable population groups. The main effects of Ni on human health are summarized in **Table 1**.

**Table 1:** Main effects of nickel on human health, with reference to absorption, distribution, systemic toxicity, allergenicity, genotoxicity, and potential tolerance mechanisms.

Effect	Description	References
Absorption and distribution	Ni is mainly absorbed through the gastrointestinal tract and, to a lesser extent, through the skin. Bioavailability ranges from 0.7–2.5% when ingested with food to 25–27% under fasting conditions or following intake through drinking water.	(Italian Ministry of Health, 2016; Schrenk et al., 2020)
Excretion	The main elimination pathway occurs through renal excretion via the kidneys and urine.	(Blanc and Moreau, 2011; Schrenk et al., 2020)
Hepatic and renal toxicity	High doses of Ni may cause liver and kidney damage, alterations in body weight, and changes in bone density and gut microbiota.	(Italian Ministry of Health, 2016)
Reproductive toxicity	May cause post-implantation loss and reduced offspring weight; in men it reduces sperm motility and alters sperm DNA, and in women it may increase the risk of abortion and fetal malformations.	(Rizvi et al., 2020; Schrenk et al., 2020)
Allergic reactions and dermatitis	Ni is among the major contact allergens and can cause allergic contact dermatitis (ACD) and SNAS, characterized by gastrointestinal and dermatological symptoms.	(Duda-Chodak and Blaszczyk, 2008; Falagiani et al., 2008)
Genotoxicity and carcinogenicity	Ni interferes with DNA repair mechanisms and increases oxidative stress, promoting genetic mutations. Ni compounds are classified as Group 1 carcinogens by IARC for lung and sinus cancers.	(Buxton et al., 2019; Das et al., 2019; Genchi et al., 2020)
Mechanisms of tolerance and desensitization	Some studies suggest that immune tolerance to Ni can be induced through controlled oral administration.	(Duda-Chodak and Blaszczyk, 2008)

## 5 Nickel role in plant physiology

**Figure 3:**
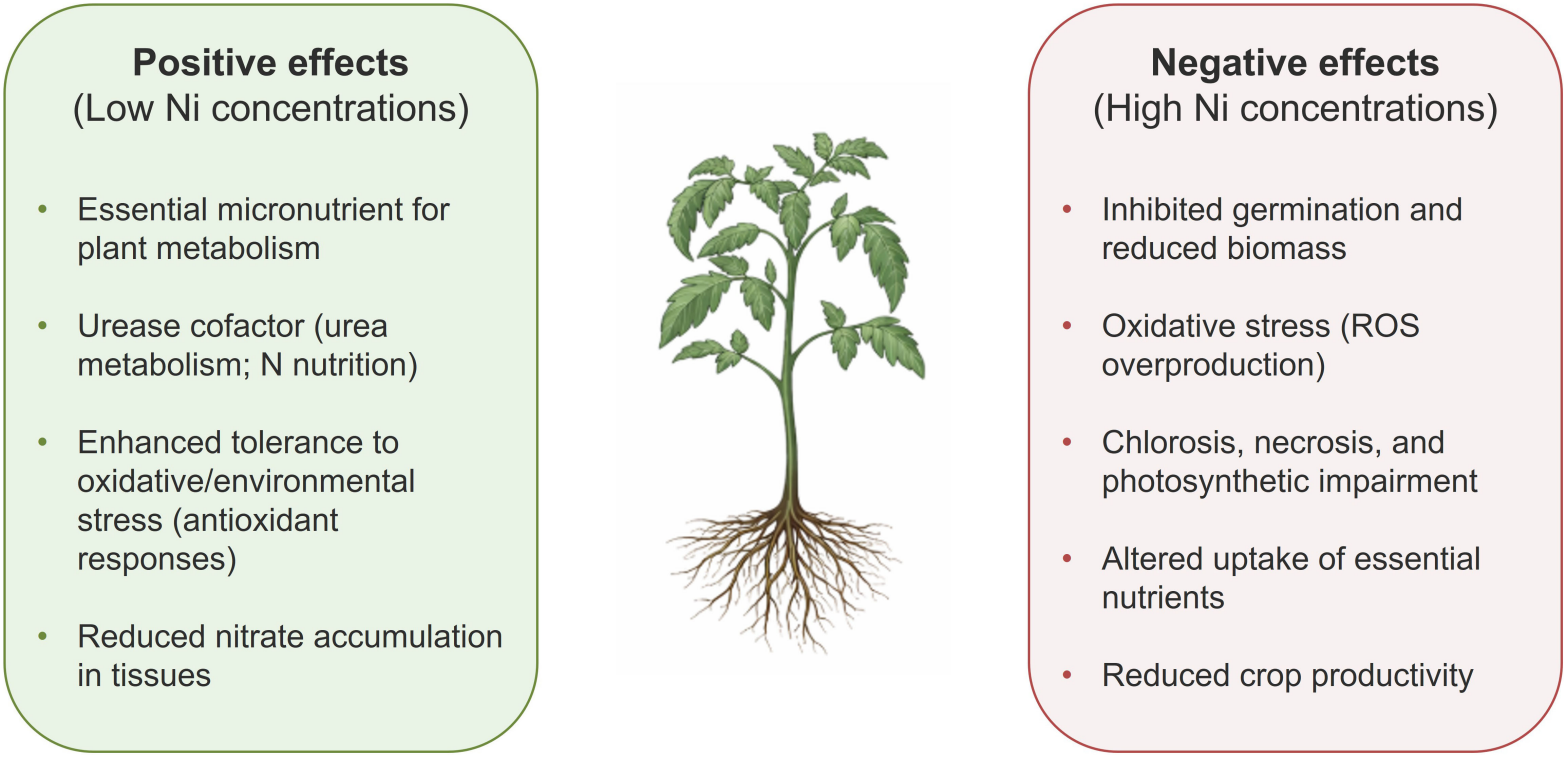
Dual role of nickel in plants as a function of tissue concentration. Adequate levels support metabolic and antioxidant functions, whereas excess levels induce oxidative stress, physiological impairment, and reduced productivity.

Ni plays a dual role in plant systems, acting as an essential micronutrient at trace concentrations while becoming toxic when present in excess. Its physiological effects depend on concentration, plant species, and environmental conditions. Ni is involved in key metabolic processes, particularly nitrogen metabolism, but elevated levels disrupt physiological and biochemical functions, ultimately affecting plant growth and productivity in **Figure 3**, indicating the narrow margin between essentiality and toxicity (Hassan et al., 2019; Shahzad et al., 2018).

Beneficial effects generally occur at tissue concentrations between 0.05 and 1 mg·kg^−1^ dry matter (DM), while levels up to about 5 mg·kg^−1^ DM may still fall within the sufficiency range depending on species. Concentrations approaching 10 mg·kg^−1^ DM represent the upper limit of adequacy and often precede the onset of toxicity (Liu et al., 2011). When this threshold is exceeded, Ni can impair photosynthesis, respiration, and enzymatic activity (Bhalerao et al., 2015; Hassan et al., 2019). However, the threshold between essentiality and toxicity is not consistently defined across plant species, and reported concentration ranges vary significantly in the literature (Hassan et al., 2019; Shahzad et al., 2018).

High concentrations induce oxidative stress through the accumulation of reactive oxygen species (ROS), which damage cellular components such as proteins, lipids, and DNA (Kováčik and Vydra, 2024). Visible symptoms often include chlorosis and leaf necrosis (Sreekanth et al., 2013).

Despite these effects, plants can tolerate moderate Ni levels through specific detoxification mechanisms, discussed in the following section. Some hyperaccumulator species, such as *A. bertolonii* and *A. argenteum All.*, are able to accumulate large quantities of Ni without severe physiological damage, making them suitable candidates for phytoremediation of contaminated soils (Chen et al., 2009; Gambi et al., 1979).

Increasing Ni concentrations in agricultural environments, largely driven by anthropogenic activities such as fertilizer use and industrial emissions, represent a growing challenge for sustainable crop production (Shahzad et al., 2018; Yang et al., 1996).

### 5.1 Uptake, transport and distribution of nickel in plants

Ni uptake occurs primarily through the roots via both passive diffusion and active transport mechanisms. Uptake efficiency depends on Ni availability in soil or NS in soilless systems, pH, and interactions with other nutrients (Marschner, 1995). Microbial processes may also play a role in regulating the mobility and speciation of Ni, as soil microorganisms can promote the mobilization of metals through mechanisms such as the production of organic ligands, redox transformations, and mineral dissolution, while Ni itself can influence the structure and activity of microbial communities (Gadd, 2010; Rizwan et al., 2024) Initial absorption takes place mainly in root hairs, where Ni^2+^ ions cross membranes following concentration gradients or through specific transport proteins regulating intracellular metal homeostasis. These transport systems include members of the ZIP (Zrt/Irt-like Protein) and NRAMP (Natural Resistance-Associated Macrophage Protein) families, which are involved in the uptake of divalent metal ions such as Fe^2+^ and Zn^2+^ (He et al., 2012; Mustafa et al., 2023). Due to the limited specificity of these transporters, Ni uptake is strongly influenced by interactions with other metals. In particular, Zn and Fe can compete with Ni for absorption sites at the root level, potentially reducing its uptake, while high concentrations of Ni can in turn disrupt the homeostasis of these essential micronutrients. Furthermore, Ni uptake occurs through both passive and active processes, involving symplastic and apoplastic pathways (He et al., 2012).

The predominant form of Ni in soil solution, typically the hydrated ion [Ni(H2O)6]^2+^, strongly influences its mobility and bioavailability (Hassan et al., 2019; He et al., 2012; Rai et al., 2019). After uptake, Ni may remain in root tissues or be translocated to aerial organs through the xylem. During transport, Ni commonly forms complexes with organic acids such as citrate and malate, which facilitate its mobility and reduce its toxicity (Cataldo et al., 1978).

Ni distribution varies among plant species. In hyperaccumulator plants such as *A. bertolonii*, Ni is efficiently translocated to the shoots and stored in leaf vacuoles. In most non-hyperaccumulator species, however, Ni remains largely confined to roots (Gambi et al., 1979; Yang et al., 1996). Redistribution to reproductive organs may also occur during plant senescence.

Interactions with other mineral elements influence Ni uptake and transport. High Ca concentrations can inhibit Ni uptake, whereas Fe and Mg may compete for transport sites in root tissues. Organic ligands and natural chelators also affect Ni mobility within plant tissues (Cataldo et al., 1978).

Understanding these processes is essential for developing strategies aimed at limiting Ni accumulation in crops.

### 5.2 Physiological function of nickel in plants

Although required only in trace amounts, Ni plays important roles in plant metabolism (Seregin and Kozhevnikova, 2006). Its most well-established function is as a structural component of urease, the enzyme responsible for urea hydrolysis. In legumes, Ni also contributes to biological nitrogen fixation and prevents metabolic disorders associated with ammonia accumulation (Liu et al., 2011; Seregin and Kozhevnikova, 2006). This reaction prevents the accumulation of toxic urea levels in plant tissues. Ni deficiency can therefore lead to metabolic disorders and symptoms such as leaf tip necrosis, observed in crops including soybean, wheat, and tomato (Dixon et al., 1975; Gerendás, 1999).

Ni also contributes to nitrogen assimilation by influencing enzymes such as nitrate reductase and glutamine synthetase. In crops such as onion and lettuce, low Ni concentrations have been shown to reduce nitrate accumulation in tissues, potentially improving product quality (Khoshgoftarmanesh et al., 2011; Stasinos et al., 2014).

In addition, Ni may enhance tolerance to environmental stress by stimulating antioxidant systems, including enzymes such as superoxide dismutase (SOD) and peroxidase (POD), which limit oxidative damage caused by ROS (Atta-Aly, 1999; Palacios et al., 1998; Scanlan et al., 1981).

Overall, Ni contributes to nitrogen metabolism, oxidative balance, and plant stress responses, highlighting its importance for normal plant growth (**Table 2**).

**Table 2:** Positive effects of nickel on plant growth and physiology, highlighting its role in nitrogen metabolism, enzymatic activation, nitrate reduction, and enhancement of plant tolerance to environmental and oxidative stresses.

Positive effect	Description	Examples of plants	References
Role in nitrogen metabolism	Ni is an essential cofactor of urease, the enzyme responsible for converting urea into ammonia and CO_2_, preventing toxic accumulation of urea in plant tissues.	Soybean, wheat, tomato	(Dixon et al., 1975; Gerendás, 1999; Seregin and Kozhevnikova, 2006)
Activation of key enzymes in nitrogen metabolism	Stimulates enzymes such as nitrate reductase and glutamine synthetase, enhancing the conversion of nitrates into plant-available nitrogen forms.	Onion, lettuce	(Khoshgoftarmanesh et al., 2011; Stasinos et al., 2014)
Reduction of nitrate accumulation in tissues	Low Ni concentrations reduce nitrate accumulation in plant tissues, potentially improving product quality and safety.	Onion, lettuce	(Khoshgoftarmanesh et al., 2011; Stasinos et al., 2014)
Improved resistance to environmental stresses	Activation of antioxidant enzymes (e.g., superoxide dismutase and peroxidases) limits the accumulation of reactive oxygen species (ROS), improving plant resistance to environmental stresses.	Cereal	(Eskew et al., 1983; Palacios et al., 1998; Atta-Aly, 1999)

### 5.3 Nickel phytotoxicity and tolerance mechanisms in plants

When present at elevated concentrations, often ranging from approximately 0.1 to 10 mM in the NS, Ni disrupts multiple physiological processes, inhibiting seed germination and impairing plant growth (Seregin and Kozhevnikova, 2006). Typical symptoms include inhibition of root and shoot elongation and tissue chlorosis or necrosis (Bhalerao et al., 2015; Shahzad et al., 2018). Roots typically accumulate higher Ni concentrations than shoots and therefore represent the main sites of toxicity (Seregin et al., 2003).

One of the principal mechanisms underlying Ni toxicity is oxidative stress. Although Ni is not redox-active, it disturbs cellular homeostasis and promotes the accumulation of ROS, including superoxide anions and hydrogen peroxide. These molecules cause lipid peroxidation, protein oxidation, and DNA damage (Boominathan and Doran, 2002). Elevated malondialdehyde (MDA) levels are frequently observed in Ni-stressed tissues (Gajewska et al., 2006).

**Figure 4:**
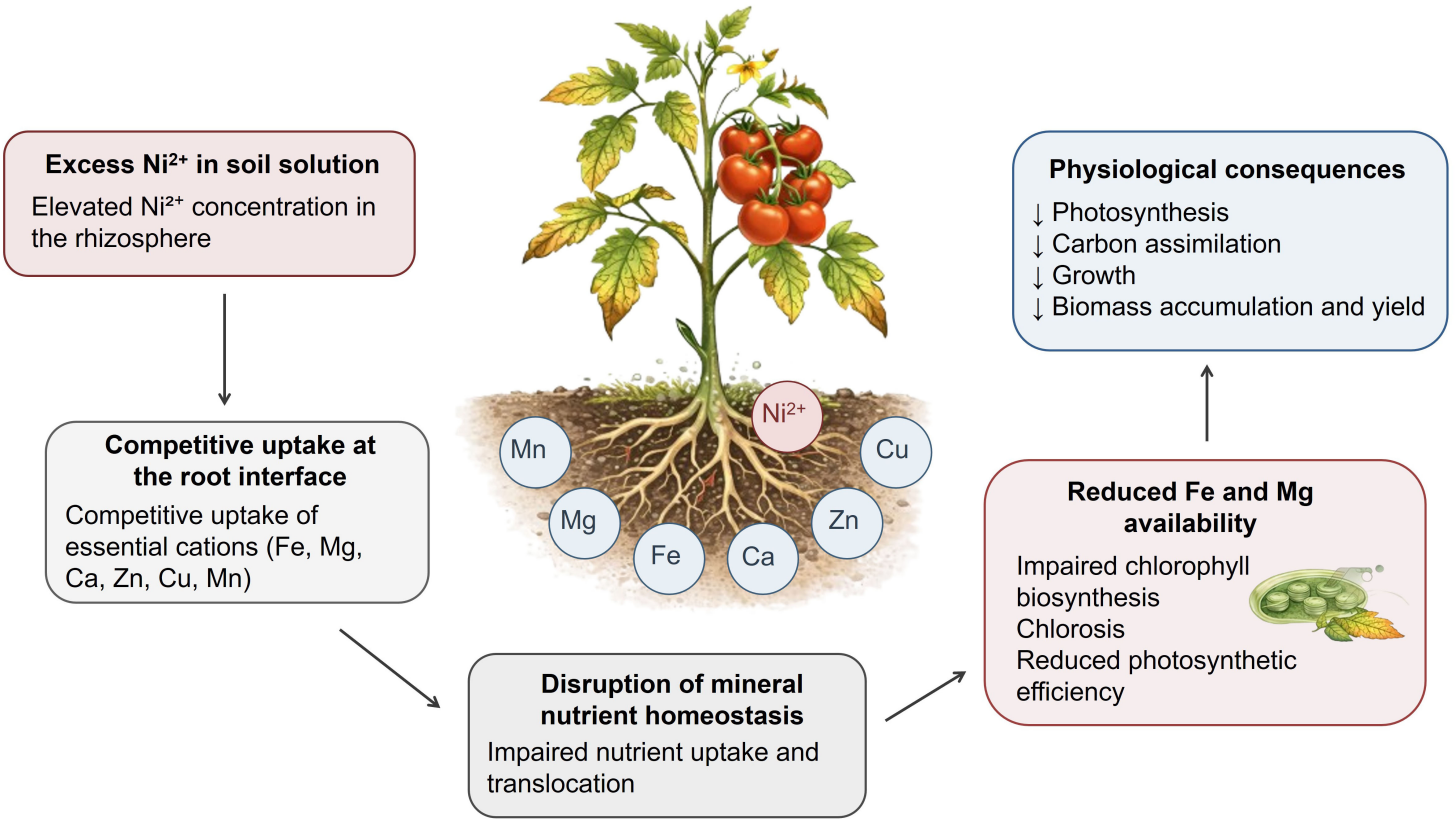
Conceptual model of nickel-induced mineral imbalance and physiological impairment in plants.

Ni excess also interferes with mineral nutrition through competition with essential nutrients such as Fe, Mg, Ca, Zn, Cu, and Mn (Gerendás, 1999; Seregin and Ivanov, 2001) (**Figure 4**), where nutrient interactions contribute to toxicity expression. Reduced Fe availability limits chlorophyll synthesis, while Mg deficiency destabilizes the photosynthetic apparatus (Pandey and Sharma, 2002). These effects are intensified under acidic conditions that increase Ni solubility (Chen et al., 2009).

Photosynthetic processes are particularly sensitive to Ni excess. High concentrations reduce photosynthesis and transpiration, partly through interference with stomatal function, and may also impair chlorophyll content, thylakoid membrane integrity, photosystems I and II, electron transport efficiency, and ATP synthesis (Bazzaz et al., 1974; Piccini and Malavolta, 1992; Singh et al., 1989). As a result, crops such as wheat and maize show significant reductions in photosynthetic performance and biomass accumulation (Bhalerao et al., 2015; Gajewska et al., 2006).

Overall, Ni phytotoxicity results from the combined effects of oxidative damage, nutrient imbalance, and photosynthetic disruption. Despite these detrimental effects, plants have evolved several physiological and biochemical mechanisms to tolerate elevated Ni concentrations and reduce metal toxicity. A key strategy involves intracellular compartmentalization, whereby Ni is sequestered into vacuoles as complexes with organic acids such as citrate and malate, thereby limiting its cytosolic reactivity (Krämer et al., 2007; Montargès-Pelletier et al., 2008). The cell wall can also bind Ni and restrict its intracellular mobility (Mustafa et al., 2023; Yusuf et al., 2011).

Chelation by intracellular ligands represents another important detoxification pathway. Ni forms stable complexes with molecules such as phytochelatins (PCs), metallothioneins (MTs), nicotianamine (NA), and glutathione (GSH), reducing the activity of free Ni^2+^ ions and facilitating sequestration (Anjum et al., 2015; Cobbett and Goldsbrough, 2002).

Plants also activate antioxidant defense systems to counteract oxidative stress associated with Ni accumulation. These include enzymatic antioxidants such as superoxide dismutase (SOD), catalase (CAT), peroxidases (POD), and ascorbate peroxidase (APX), together with non-enzymatic compounds such as ascorbate and tocopherol (Sachan and Lal, 2019; Wu et al., 2010).

Hormonal signaling further contributes to stress adaptation. Abscisic acid (ABA) regulates stomatal conductance and water balance, while salicylic acid, ethylene, and auxins participate in defense responses and redox regulation (Gajewska and Skłodowska, 2005; Molas, 1997).

Certain hyperaccumulator species exhibit exceptional tolerance, accumulating more than 1,000 mg Ni·kg^−1^ dry weight without visible toxicity symptoms. Examples include A. bertolonii, Thlaspi caerulescens J. Presl & C. Presl, and Phyllanthus spp. L., which possess specialized transport systems that enhance Ni uptake, translocation to shoots, and vacuolar sequestration (Deng et al., 2018; Verbruggen et al., 2009).

Environmental factors such as soil pH, salinity, organic matter content, and interactions with other metals can strongly influence Ni bioavailability and plant responses (Ameen et al., 2019; Gabbrielli et al., 1991). Ni tolerance therefore results from the coordinated action of sequestration, chelation, antioxidant defenses, and physiological regulation.

## 6 Increased interests in vegetables and “Ni-free” foods

In recent years, there has been a growing focus on foods and vegetables with reduced Ni content, often marketed as “Ni-free”, as a direct response to the increase in diagnoses of SNAS and contact dermatitis related to dietary intake of the metal. This new sensitivity is reflected not only in scientific literature and increased public awareness, but also in the concrete transformation of the agrifood supply and organized distribution, with the increasingly frequent appearance of dedicated spaces in large-scale retail outlets.

**Figure 5:**
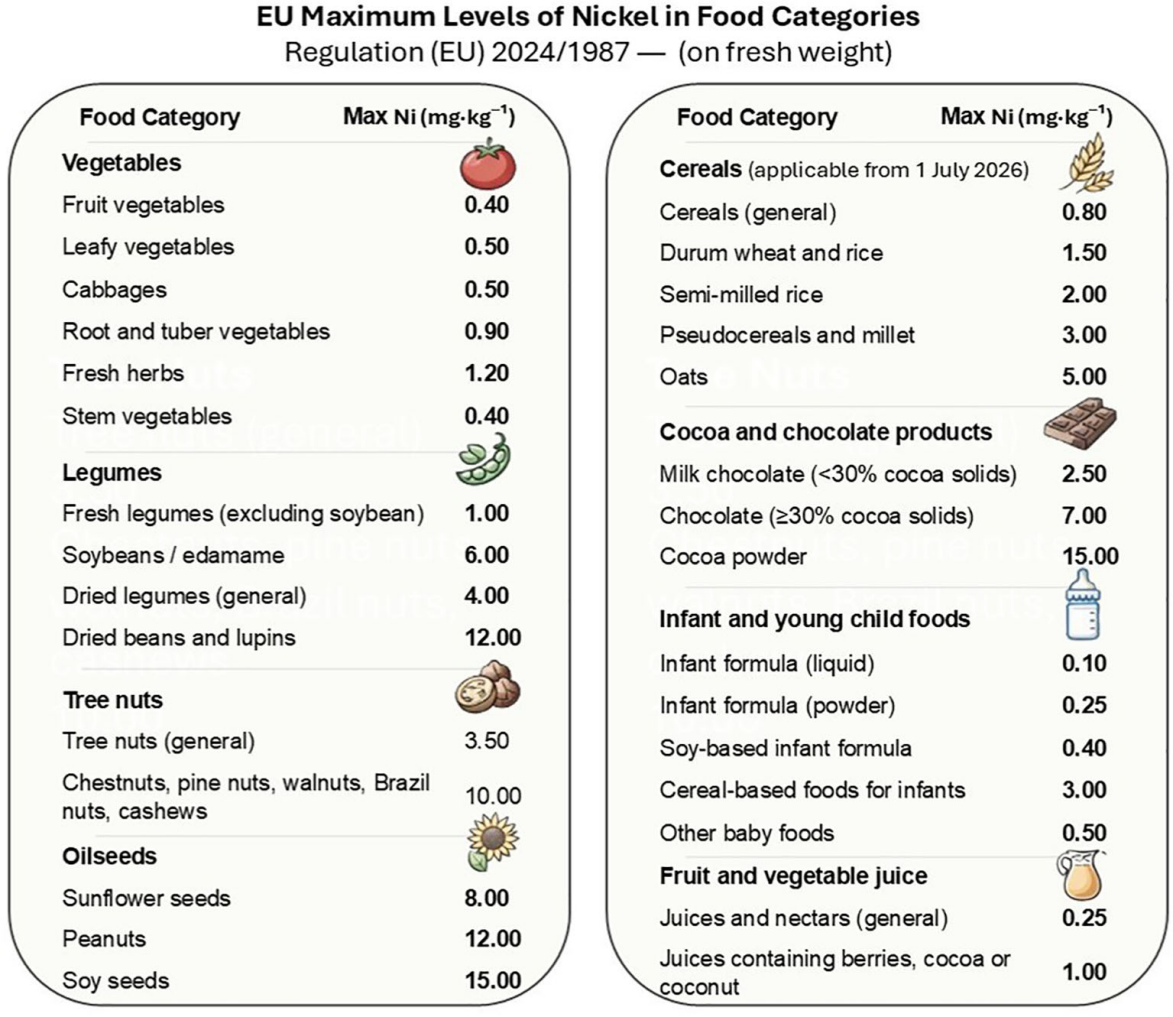
Overview of maximum nickel levels established by Commission Regulation (EU) 2024/1987 across major food groups, highlighting category-specific limits.

The growing demand for low-Ni foods has also been reinforced by recent regulatory developments. In 2024, the European Union adopted Commission Regulation (EU) 2024/1987, which introduced maximum levels of Ni in several food categories, including cereals, legumes, vegetables, nuts, and baby food (**Figure 5**), reflecting differentiated regulatory thresholds across food groups. These limits will progressively apply from 2025 onwards and aim to reduce chronic dietary exposure to Ni, particularly in sensitive population groups (European Commission, 2024a). However, the growing diffusion of “Ni-free” products reflects increasing demand from Ni-sensitive consumers, particularly individuals affected by SNAS.

### 6.1 Impact on agricultural production and the food industry

In response to the increasing demand for “Ni-free” products, the agricultural sector has implemented mitigation strategies aimed at limiting Ni accumulation in crops. These approaches include the adoption of soilless cultivation systems and stricter control of agronomic practices to reduce metal bioavailability. Concurrently, the food industry has introduced products marketed as “Ni-free”, with batch verification performed through analytical techniques such as Inductively Coupled Plasma Mass Spectrometry (ICP-MS) or Atomic Absorption Spectroscopy (AAS) to substantiate labelling claims. Recent EU legislation has strengthened the analytical framework for Ni determination in foodstuffs. Commission Implementing Regulation (EU) 2024/1045 introduced specific requirements for sampling procedures and analytical performance criteria for the official determination of Ni in foods, ensuring reliable quantification at trace concentration levels (Regulation EU 2024/1045) (European Commission, 2024b).

However, the designation “Ni-free” raises important regulatory concerns. Within the European Union, voluntary food labeling is governed by Regulation (EU) No 1169/2011 and Regulation (EC) No 1924/2006. However, neither regulation provides a harmonized legal definition or analytical threshold for the use of “Ni-free” or “Ni-tested” claims European Parliament and Council of the European Union, 2006; European Parliament and Council of the European Union, 2011. In commercial practice, the term “Ni-free” is often used to indicate Ni concentrations below the analytical detection or quantification limit of the method applied, whereas the designation “Ni-tested” generally refers to products analytically verified to contain Ni below a declared threshold. In the absence of an official threshold, the term lacks standardized legal value, and analytical criteria are based on voluntary benchmarks, leading to inconsistencies among products and producers.

A further challenge lies in the intrinsic variability of Ni content in plant-derived foods, even under controlled soilless systems. Ni accumulation in plants is influenced by plant genotype, water quality, substrate composition, NS chemistry, interactions with competing ions, and pH conditions. In closed-cycle hydroponic systems, cumulative Ni inputs from fertilizers may progressively increase Ni concentration in the NS, enhancing the risk of uptake, translocation, and accumulation in plant tissues. Therefore, targeted research is required to develop effective mitigation strategies in controlled cultivation systems. Priorities include the optimization of fertilizer composition, implementation of NS de-nickelization protocols, careful selection of substrates and irrigation water, and breeding or selection of genotypes with reduced Ni accumulation capacity. The establishment of standardized thresholds and production protocols is essential to ensure consistency, transparency, and scientific robustness within the emerging “Ni-free” supply chain.

### 6.2 The limits of current “Ni-free” voluntary certification

From a regulatory and analytical perspective, the term “Ni-free” does not indicate the complete absence of Ni but rather refers to products containing Ni concentrations below voluntarily adopted thresholds that are not harmonized at the European level. From a strictly scientific perspective, the term “Ni-free” is intrinsically misleading, as Ni is a ubiquitous trace element in environmental and biological systems and its complete absence in food matrices is virtually unattainable. In practice, the claim “Ni-free” generally refers to Ni concentrations below the analytical detection or quantification limit of the method used. Conversely, the designation “Ni-tested” indicates products whose Ni content has been analytically determined and verified to remain below a declared threshold used for product qualification. Despite the increasing availability of “Ni-free” products on the market, no official regulation currently defines standardized criteria for the use of this claim.

**Figure 6:**
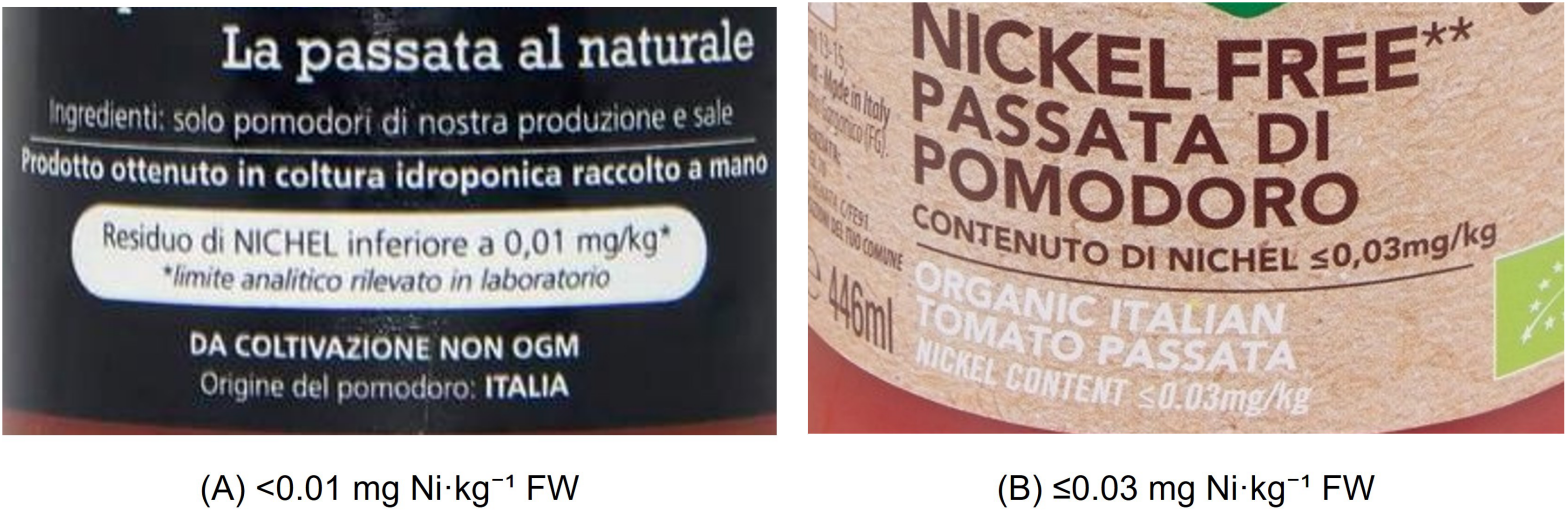
Examples of voluntary “Ni-free” labeling in commercial tomato purée products available in large-scale retail distribution. **(A)** Label reporting “Residuo di Nichel inferiore a 0,01 mg/kg – limite analitico rilevato in laboratorio” (“Nickel residue lower than 0.01 mg kg^−1^ – analytical limit determined in laboratory”). **(B)** Label reporting “Nickel free – contenuto di nichel ≤0,03 mg/kg” (“Nickel-free – nickel content ≤0.03 mg kg^−1^”). The thresholds declared on the labels are voluntarily adopted by manufacturers and are not based on harmonized European regulatory criteria.

The main limitations of the current certification framework can be summarized as follows:

- Absence of regulated thresholds: there is no legally established maximum Ni concentration below which a food product may be officially classified as “Ni-free”.
- Lack of technical guidelines: no harmonized standards exist regarding sampling procedures, analytical frequency, verification protocols, or labeling requirements.
- Limited public oversight: although analytical testing (e.g., ICP-MS or AAS) is often performed to support the claim, results are not systematically subject to independent third-party verification or public disclosure.
- Non-systematic sampling: controls are typically conducted on representative batches rather than on the entire production, potentially leading to inter-lot variability.
- Unregulated claim usage: in the absence of binding legislation, the “Ni-free” designation may be applied without standardized certification or transparency obligations. This heterogeneity in declared threshold values is illustrated in (**Figure 6**), showing the lack of consistency in applied thresholds.

These regulatory gaps introduce significant variability and discretion into the system, potentially being misleading for consumers, as it may be interpreted as the complete absence of Ni rather than concentrations below analytical thresholds, thereby undermining transparency, traceability, and market consistency The establishment of clear, harmonized, and scientifically justified criteria for “Ni-free” labeling therefore appears urgent in order to ensure transparency, comparability among products, and credibility within the supply chain. It therefore appears essential to establish clear thresholds and analytical frameworks, harmonized to ensure transparency, comparability between products and credibility within the supply chain.

### 6.3 The diffusion of “Ni-free” products in large-scale distribution (GDO)

**Figure 7:**
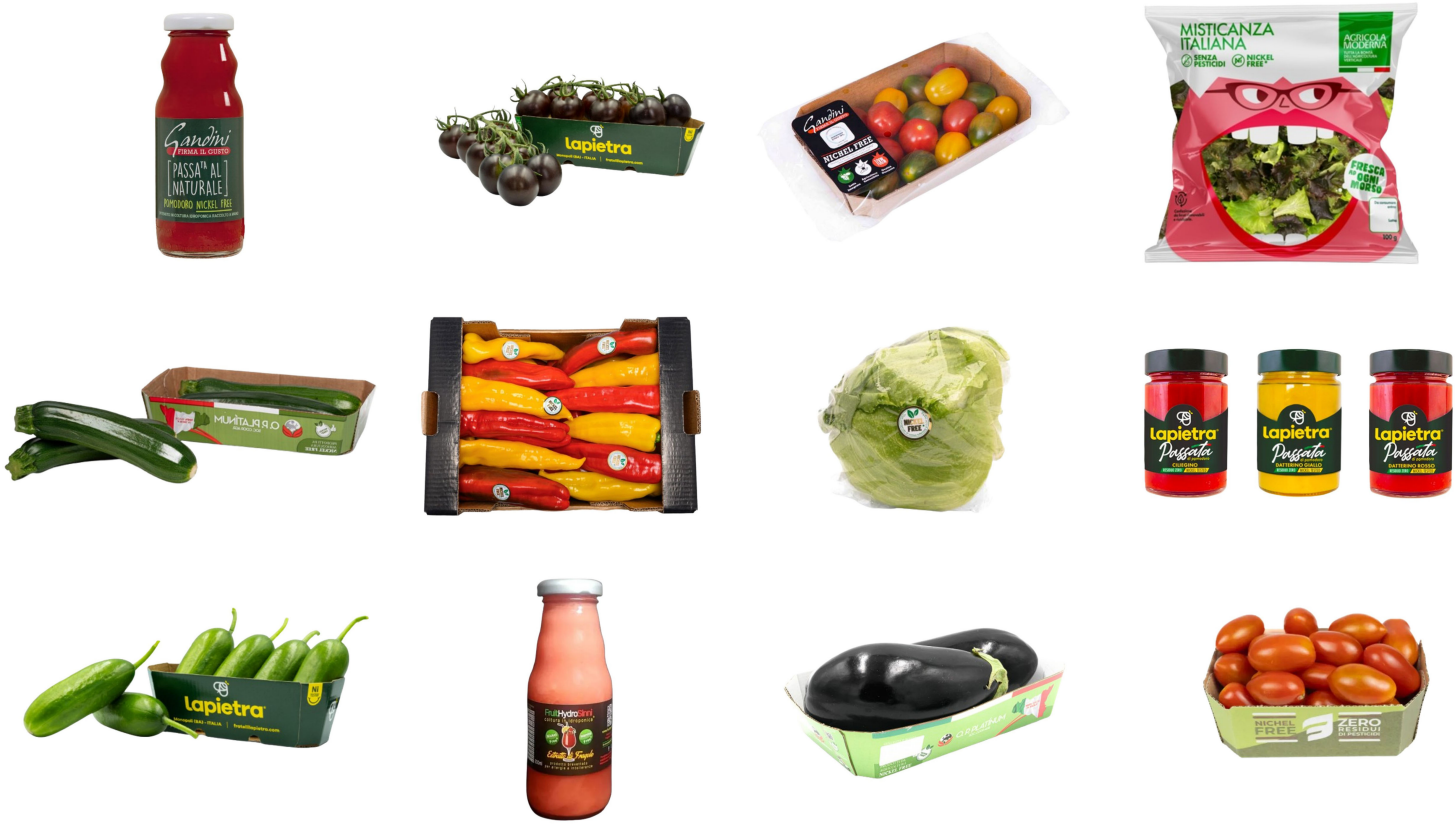
Retail diversification of “Ni-free” food products in large-scale distribution (GDO). Product images were collected from the official websites of the respective producers and retailers for illustrative purposes. (https://fratellilapietra.it/; https://www.pomodorigandini.it/it/; https://agricolamoderna.com/; https://www.opplatinumshop.it/).

In recent years, the European food market has experienced sustained growth in the “free-from” segment (e.g., gluten-free, lactose-free, “Ni-free”), reflecting increasing consumer demand for products tailored to specific dietary sensitivities and creating new opportunities for product differentiation. Within this context, demand from individuals affected by SNAS or documented Ni sensitivity has stimulated the expansion of “Ni-free” product lines in large-scale retail distribution. Retail chains have progressively incorporated “Ni-free” labelled products, increasing their visibility and accessibility through dedicated shelf spaces and targeted instore information (**Figure 7**).

At the production level, several farms, particularly those adopting soilless cultivation systems, have implemented agronomic practices aimed at minimizing Ni accumulation in vegetables, leading to the development of specialized supply chains based on controlled production systems and analytical verification.

However, despite this market expansion, differences in analytical thresholds and certification practices among producers may lead to variability in the interpretation of “Ni-free” products. Divergent analytical thresholds and heterogeneous certification practices may compromise transparency and undermine consumer confidence. Although the introduction of maximum Ni limits under Regulation (EU) 2024/1987 has strengthened food safety standards, these thresholds are designed to protect the general population and do not specifically address the expectations of Ni-sensitive individuals seeking products with concentrations significantly below the established maximum levels.

For this segment to develop in a credible and sustainable manner, the establishment of clear and harmonized regulatory criteria appears essential to ensure consistency, traceability, and consumer protection.

## 7 Agronomic inputs as primary determinants of nickel content in soilless cultivation systems

In soilless cultivation systems, particularly in closed-cycle systems with NS recirculation, the presence of Ni is closely related to the agronomic inputs introduced into the system. Unlike open-field crops, where Ni content mainly reflects soil geochemistry and weathering processes, in closed cycle soilless systems metal concentration can be reconstructed through a quantitative analysis of technical sources: fertilizers, plant protection products, substrates, and feedwater. This configuration makes possible a mass balance approach, aimed at identifying the hierarchy of sources and defining the main agronomic control points. In this context, the data presented in the following sections are intended to constitute an illustrative case study, aimed at exemplifying the application of a mass balance approach under farm conditions and supporting the identification of key agronomic control points. This aspect takes on particular relevance in the context of the production of “Ni-free” vegetables, where even trace inputs can influence the final concentration in plant tissues.

The following paragraphs present data collected by the farm Azienda Agricola F.lli Lapietra (https://fratellilapietra.it/) concerning the analysis of Ni content in the main production inputs used for the soilless cultivation of tomato and cucumber, including fertilizers, irrigation water, plant protection products, and growing substrates. The presentation of these data aims to highlight how Ni is a trace metal that can occur in most agricultural matrices and may therefore enter the production cycle through multiple inputs. This exploratory assessment represents the starting point for ongoing research activities carried out by the farm to develop production strategies aimed at obtaining vegetables with controlled Ni content.

### 7.1 Mineral fertilizers: analytical characterization and contribution to the nutrient solution

Within the experimental activities conducted at farm level, ICP-MS analyses were performed by Euroquality Lab (Gioia del Colle, BA, Italy – Accredia n. 00187) on the fertilizers used for the preparation of NS applied for tomato cultivation in the studied hydroponic system, revealing the systematic presence of Ni as a technological impurity. The measured concentrations (mg Ni·kg^−1^) are reported in **Table 3**, showing marked variability among fertilizer matrices, with differences exceeding two orders of magnitude, mainly reflecting variations in raw materials and manufacturing processes.

**Table 3:** Nickel concentrations in commercial fertilizers used for nutrient solution preparation, determined by ICP-MS.

Chemical formula	Fertilizer (commercial name)	Manufacturer	Nickel (mg Ni·kg^−1^)
Ca(NO_3_)_2_	Calcinit^®^	Yara International	10.417
KNO_3_	Multi-K^®^ G.G.	Haifa Group	0.266
NH_4_NO_3_	Amnitra^®^	Yara International	0.233
Mg(NO_3_) _2_	Magnisal^®^	Haifa Group	0.120
CaCl_2_	Calcium Chloride Flakes	TETRA Technologies	28.84
KCl	Haifa MOP™	Haifa Group	0.057
MgSO_4_·7H_2_O	Bittermag^®^	K+S Minerals	0.578
KH_2_PO_4_	Haifa MKP™	Haifa Group	0.143
K_2_SO_4_	Haifa SOP™ Prime	Haifa Group	25.794
MnSO_4_	Haifa Micro™ Mn	Haifa Group	80.481
H_3_BO_3_	Haifa Micro™ B	Haifa Group	0.054
Cu-EDTA	Haifa Micro™ Cu	Haifa Group	4.222
Na_2_MoO_4_	Haifa Micro™ Mo	Haifa Group	0.017
Fe-DTPA	Haifa Micro™ Fe-DTPA	Haifa Group	9.371

However, the Ni concentration in the commercial fertilizer does not directly correspond to its effective contribution to the NS. In fertigation systems, the relevant parameter is the total Ni load introduced into the system, which depends on the Ni concentration in the fertilizer, the amount applied in the fertigation system, and the dilution factor used to prepare the final NS.

To estimate the operational concentration, the preparation process of the fertigation mixture was reconstructed. The NS contained (mg·L^-1^): 230 total N, including 206 N-NO_3_^−^ and 24 N-NH_4_, 79 P, 380 K, 250 Ca, 58 Mg, 160 S and 100 Cl, whereas micronutrient concentrations were 2.4 Fe, 1.1 Mn, 0.2 Zn, 0.49 B, 0.075 Cu and 0.078 Mo. For each fertilizer, the Ni input was calculated by multiplying the measured Ni concentration (mg Ni·kg^−1^) by the mass of fertilizer dissolved in the 1000 L stock tank and then applying the dilution factor to obtain the final concentration in the NS. The overall Ni concentration was obtained by summing the individual contributions.

**Figure 8:**
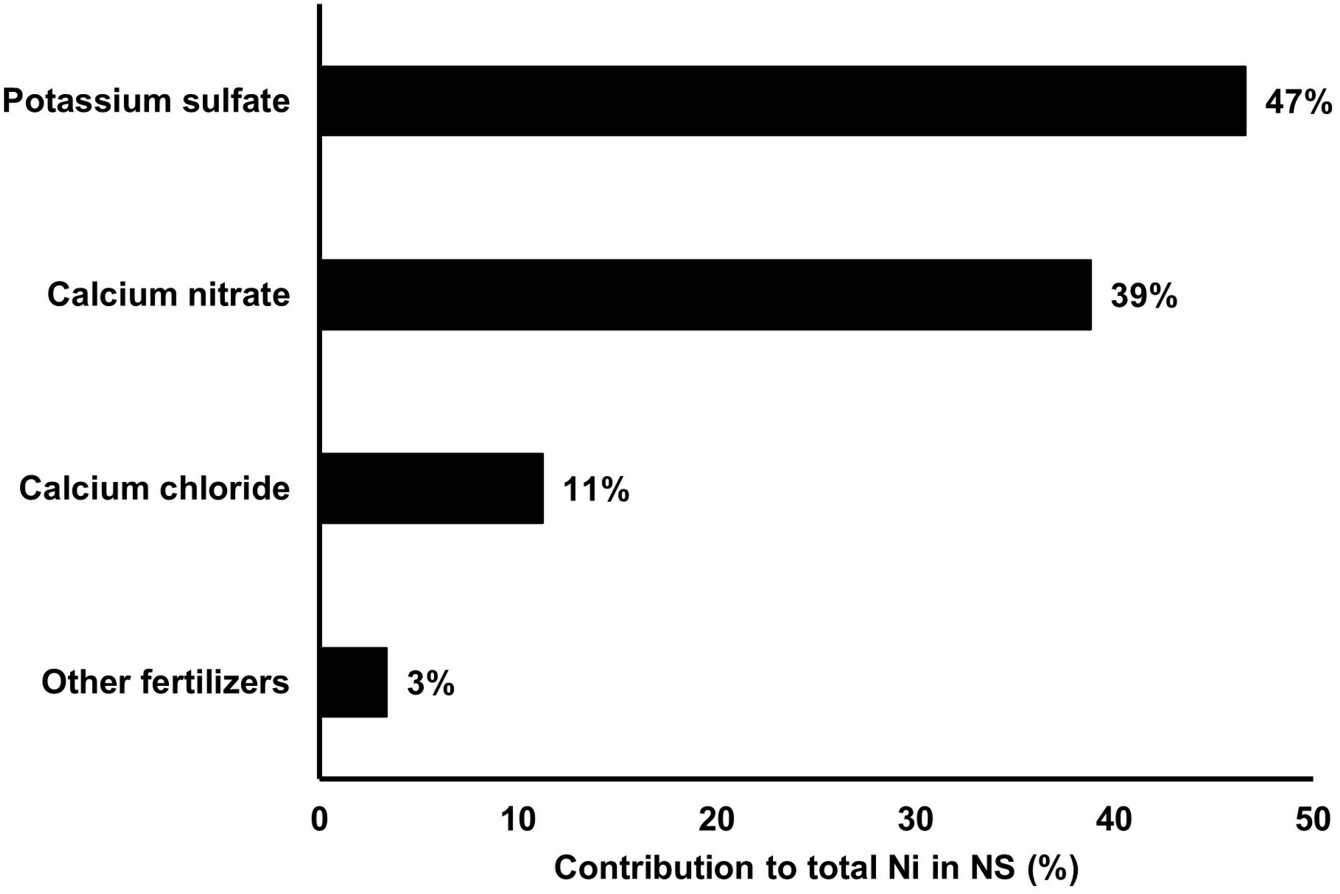
Relative contribution of mineral fertilizers to the total Ni load in the nutrient solution of the monitored commercial soilless tomato and cucumber production system. Percentages were calculated based on a mass balance reconstruction using Ni concentrations measured in this study by ICP-MS and the actual fertilizer application rates. The main fertilizers included potassium sulfate (K_2_SO_4_, Haifa SOPTM Prime, Haifa Group), calcium nitrate (Ca(NO_3_)_2_, Calcinit^®^, Yara International) and calcium chloride (CaCl_2_, calcium chloride flakes, TETRA Technologies).

The model estimated a theoretical Ni concentration in the NS between 0.032 and 0.036 mg Ni·L^−1^ (32–36 µg Ni·L^−1^). Contribution analysis showed that potassium sulfate accounted for approximately 47% of the total Ni load, followed by calcium nitrate (≈39%) and calcium chloride (≈11%), while all other fertilizers individually contributed less than 3%. The relative contribution of the different fertilizers to the total Ni load in the NS is shown in **Figure 8**, where few fertilizers dominate total Ni input. Overall, more than 86% of the Ni in the NS originated from two widely used fertilizers, potassium sulfate (K_2_SO_4_) and calcium nitrate (Ca(NO_3_)_2_). Their dominant contribution is primarily related to the large quantities applied in the fertigation system, rather than to particularly high Ni concentrations in the products themselves. To verify the reliability of the model, the NS was sampled directly from the fertigation system before distribution to tomato and cucumber plants and analyzed by ICP-MS. Replicated analyses showed an average Ni concentration of 26.1 ± 1.55 µg Ni·L^−1^, consistent with the theoretical estimate (32–36 µg Ni·L^−1^). The observed deviation (≈20–25%) is likely attributable to variability among fertilizer batches, minor operational differences in stock solution preparation, and analytical uncertainty. Irrigation water contained less than 0.001 mg Ni·L^−1^, excluding water as a relevant Ni source in the system.

### 7.2 Secondary sources: contribution of pesticides and substrates to the nickel balance

In addition to mineral fertilization, other potential pathways for Ni entry into soilless cropping systems include plant protection products and growing media. Although these matrices may contain Ni as a contaminant, quantitative analysis allows their contribution to be evaluated relative to fertilization. ICP-MS analyses were performed on the plant protection products used during the monitored commercial tomato and cucumber crop cycle. In most formulations, Ni concentrations were below the analytical limit of quantification (LOQ= 30 µg Ni·kg^−1^). Only one product (Evidance) showed a measurable concentration (5.851 mg Ni·kg^−1^). The complete analytical results are reported in **Table 4**.

**Table 4:** Nickel concentration in commercial plant protection products applied during the monitored soilless tomato and cucumber production cycle, determine by ICP-MS analysis.

Commercial product	Nickel (µg Ni·kg^−1^)	Analytical interpretation
Affirm^®^ (Syngenta Crop Protection AG, Switzerland)	< 30	Below LOQ
Agree^®^ WG (Certis Biologicals, USA)	< 30	Below LOQ
CoStar^®^ WG (Certis Biologicals, USA)	< 30	Below LOQ
Elicin^®^ BZ (CBC Europe S.r.l., Biogard Division, Italy)	< 30	Below LOQ
Eradicoat^®^ (Certis Europe B.V., Netherlands)	< 30	Below LOQ
Evidance^®^ (Sipcam Italia S.p.A., Italy)	5851	Quantified value
Flipper^®^ (Bayer CropScience AG, Germany)	< 30	Below LOQ
Frontiere^®^ (CBC Europe S.r.l., Biogard Division, Italy)	< 30	Below LOQ
Karma^®^ (Certis Europe B.V., Netherlands)	< 30	Below LOQ
NeemAzal^®^-T/S (Trifolio-M GmbH, Germany)	< 30	Below LOQ
Oberon^®^ (Bayer CropScience AG, Germany)	< 30	Below LOQ
Prev-Am^®^ (Biobest Group NV, Belgium)	< 30	Below LOQ
Tiovit^®^ Jet (Syngenta Crop Protection AG, Switzerland)	< 30	Below LOQ

Concentrations refer to formulated products analyzed as supplied. LOQ (limit of quantification) = 30 (µg Ni·kg^−1^). Values below the LOQ are reported as <30 µg Ni·kg^−1^ indicate concentrations below the analytical limit of quantification.

From an agronomic perspective, the contribution of plant protection products depends not only on their Ni concentration but also on the quantities applied and the frequency of use. Unlike fertilizers, which are fully dissolved in the NS and continuously supplied throughout the crop cycle, plant protection products are applied in much smaller quantities and with episodic frequency. Consequently, their overall contribution to the Ni balance of the system is quantitatively negligible compared with that of mineral fertilization.

The role of the growing medium was evaluated by distinguishing between the intrinsic Ni content of the virgin material and its variation during the crop cycle. ICP-MS analyses performed on rockwool samples collected in the commercial tomato greenhouse showed a total Ni content of 0.39 mg Ni·kg^−1^ in the virgin substrate. During the crop cycle, Ni concentration increased to 3.7 ± 0.97 mg Ni·kg^−1^ and reached 21.0 ± 2.6 mg Ni·kg^−1^ at the end of the cycle. This progressive increase indicates accumulation over time, consistent with the continuous supply of Ni through the NS and with retention processes occurring on the substrate surfaces.

These results indicate that the substrate does not represent an intrinsic source of Ni but rather acts as a passive storage compartment in which the metal supplied through fertigation can accumulate. The observed increase reflects the balance between repeated inputs from the NS and outputs due to root uptake or possible leaching.

**Figure 9:**
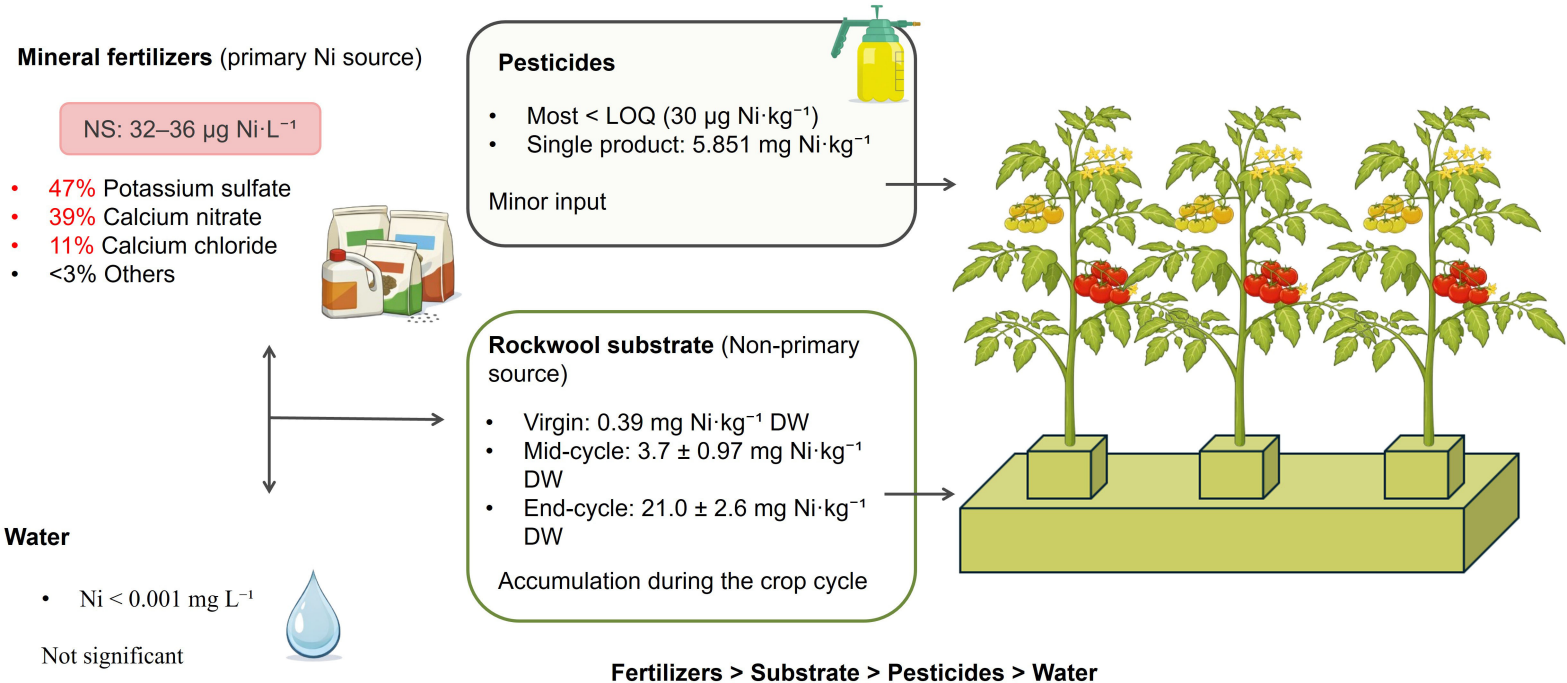
Conceptual Ni mass balance in a closed-loop soilless cultivation system.

Overall, the integrated analysis conducted in the commercial soilless tomato and cucumber production system shows that Ni is primarily introduced through mineral fertilizers, while plant protection products and substrates play a secondary role. Consequently, the final Ni content in the crop is largely determined by the quality and composition of the fertilizers used. Analytical control of fertilization inputs therefore represents the main management strategy for limiting Ni accumulation in soilless systems. The relative contribution of the different Ni sources and the role of the substrate as an accumulation compartment are summarized in **Figure 9**, supporting a system-level interpretation of Ni inputs. Taken together, these findings support a more integrated interpretation of Ni transfer across agri-food systems. While environmental and technical sources determine the initial Ni input, plant uptake represents the key biological step linking these inputs to edible tissues and, ultimately, to human exposure. In soil-based systems, Ni accumulation is largely governed by geochemical background and bioavailability, whereas in closed-cycle soilless systems the main control points shift toward the management of agronomic inputs. This distinction is particularly relevant for the development of reliable Ni-reduced, “Ni-free”, or “Ni-tested” production systems, highlighting that effective mitigation strategies must act upstream, through the control of inputs entering the cultivation system.

## 8 Future perspectives and research directions

Despite the extensive scientific literature on Ni dynamics in the environment and along the food chain, several knowledge gaps still limit a precise assessment of its impact on plant systems and human health. One of the most critical unresolved issues concerns the effective bioavailability of dietary Ni, which depends not only on total metal concentration but also on chemical speciation, food matrix composition, processing methods, and interactions with other dietary components (Genchi et al., 2020; Schrenk et al., 2020). Current exposure assessments are largely based on total Ni content and may therefore over- or underestimate actual health risks.

From an agronomic perspective, future research should focus on optimizing “Ni-free” and “Ni-tested” cultivation systems, particularly in hydroponic production. Although these systems allow tighter control of nutrient inputs compared with open-field cultivation, Ni may still enter the production chain through fertilizers, irrigation water, or NS recirculation (Hassan et al., 2019; He et al., 2012). In this context, the development of selective chelating agents with high affinity for Ni could represent a promising strategy to reduce its bioavailable fraction in NS while preserving the availability of essential micronutrients. In parallel, the establishment of standardized protocols for NS management, substrate selection, and fertilizer formulation would contribute to improving the stability and reproducibility of “Ni-free” horticultural production.

At the plant level, increasing attention should be directed toward genotype-dependent differences in Ni uptake, transport, and compartmentalization. Several studies have shown that plant species and cultivars differ significantly in their capacity to accumulate and tolerate Ni through mechanisms such as vacuolar sequestration, chelation, and enhanced antioxidant activity (Chen et al., 2009; Yang et al., 1996). Advances in plant breeding and biotechnology may therefore support the selection of cultivars with reduced Ni accumulation without compromising yield or nutritional quality.

From a toxicological and clinical perspective, further studies are needed to clarify the dose–response relationship of Ni in sensitized individuals, particularly those affected by SNAS. Current dietary recommendations remain largely empirical and are not yet supported by robust clinical evidence (Duda-Chodak and Blaszczyk, 2008; Falagiani et al., 2008). In this context, the emerging role of the intestinal microbiota in modulating Ni absorption and immune responses represents a promising research avenue with potential implications for personalized dietary strategies (Genchi et al., 2020).

## 9 Conclusion

Ni is a dual-function element in agri-food systems, acting as both an essential micronutrient and a potential contaminant. Its accumulation in plant-based foods depends on concentration, speciation, and bioavailability, and is shaped by interacting factors such as environmental conditions, agronomic practices, and crop genotype, leading to high variability across production systems.

A key contribution of this review is the recognition of soilless cultivation, particularly closed-cycle hydroponics, as a highly controllable framework for Ni management. In these systems, Ni inputs are mainly associated with fertilizers rather than soil geochemistry, shifting control toward agronomic decisions. The evidence indicates that selecting low-Ni fertilizers and implementing systematic NS monitoring are the most effective strategies to limit Ni accumulation in edible tissues.

These findings have direct practical and health implications. While dietary Ni exposure is generally within safe limits for the overall population, it remains a concern for sensitized individuals (e.g., SNAS), increasing the demand for low-Ni foods. In this context, Regulation (EU) 2024/1987 establishes maximum Ni levels for food safety, but does not define harmonized criteria for “Ni-free” or “Ni-tested” claims, creating potential inconsistencies and reduced transparency for consumers.

Future research should focus on reducing Ni bioavailability through improved NS design, developing standardized and sensitive analytical methods, and establishing harmonized thresholds and certification schemes. An integrated approach combining agronomic control, analytical validation, and regulatory alignment is essential to support safe, transparent, and reliable low-Ni production systems.
