# Supplementary material for: Nickel in agri-food systems: a review
Source: Front Plant Sci. 2026 Jun 26;17:1829209. doi: 10.3389/fpls.2026.1829209 (PMC13350349; doi:10.3389/fpls.2026.1829209)
Supplement: Supplementary file 1 [file Table1.docx]

Table S1 Major natural and anthropogenic sources of nickel in environmental systems, including concentration data and descriptive notes, highlighting the variability associated with geochemical factors and anthropogenic inputs.

| **Source type** | **Source** | **Typical Ni concentration** | **Notes** | **References** |
| --- | --- | --- | --- | --- |
| Natural | Ultramafic rocks / serpentine soils | 500–10,000 mg·kg^-1^ | High geogenic background | (Ghaderian et al. 2007; Quantin et al. 2008) |
| Natural | Non-ultramafic soils | highly variable | Background levels depend on parent material | (Reimann and de Caritat 2012) |
| Anthropogenic | Phosphate fertilizers | up to 14.8 mg·kg-1 | Depends on phosphate rock origin and processing | (Nziguheba and Smolders 2008) |
| Anthropogenic | Industrial emissions / fossil fuel combustion | highly variable | Atmospheric deposition and local enrichment | (Cempel and Nikel 2006; Shahzad et al. 2018) |
| Anthropogenic | Urban / traffic sources | highly variable | Transport, heating, corrosion, construction/demolition particles | (Cempel and Nikel 2006; Shahzad et al. 2018) |
| Anthropogenic | Metallurgical activities | site-specific, often elevated | Point-source contamination from stainless steel and alloy production | (Cempel and Nikel 2006) |
